# In Vitro Activity of Dalbavancin against Refractory Multidrug-Resistant (MDR) *Staphylococcus aureus* Isolates

**DOI:** 10.3390/antibiotics9120865

**Published:** 2020-12-03

**Authors:** Dafne Bongiorno, Lorenzo Mattia Lazzaro, Stefania Stefani, Floriana Campanile

**Affiliations:** Department of Biomedical and Biotechnological Sciences (BIOMETEC)—Medical Molecular Microbiology and Antibiotic Resistance Laboratory (MMARLab), University of Catania, 95123 Catania, Italy; d.bongiorno@unict.it (D.B.); lazzclml@gmail.com (L.M.L.); stefania.stefani@unict.it (S.S.)

**Keywords:** *Staphylococcus aureus*, MDR-MRSA, dalbavancin, bactericidal activity, refractory clones

## Abstract

The high prevalence of methicillin-resistant *Staphylococcus aureus* (MRSA) infections, always treated with vancomycin and daptomycin, has led to the emergence of vancomycin-intermediate (VISA), heteroresistant vancomycin-intermediate (hVISA) and daptomycin non-susceptible (DNS) *S. aureus*. Even if glycopeptides and daptomycin remain the keystone for treatment of resistant *S. aureus*, the need for alternative therapies that target MRSA has now become imperative. The in vitro antibacterial and bactericidal activity of dalbavancin was evaluated against clinically relevant *S. aureus* showing raised antibiotic resistance levels, from methicillin-susceptible to Multidrug-Resistant (MDR) MRSA, including hVISA, DNS and rifampicin-resistant (RIF-R) strains. A total of 124 *S. aureus* strains were tested for dalbavancin susceptibility, by the broth microdilution method. Two VISA and 2 hVISA reference strains, as well as a vancomycin-resistant (VRSA) reference strain and a methicillin-susceptible *Staphylococcus aureus* (MSSA) reference strain, were included as controls. Time–kill curves were assayed to assess bactericidal activity. Dalbavancin demonstrated excellent in vitro antibacterial and bactericidal activity against all *S. aureus* resistance classes, including hVISA and DNS isolates. The RIF-R strains showed the highest percentage of isolates with non-susceptibility, reflecting the correlation between *rpo*B mutations and VISA/hVISA emergence. Our observations suggest that dalbavancin can be considered as an effective alternative for the management of severe MRSA infections also sustained by refractory phenotypes.

## 1. Introduction

Reduced susceptibility to glycopeptides in *Staphylococcus aureus* poses a great threat to antimicrobial chemotherapy worldwide, and particularly in methicillin-resistant *S. aureus* (MRSA), it is seriously challenging to the therapeutic field. Vancomycin-intermediate *S. aureus* strains with homogeneous (VISA) or heterogeneous (hVISA) phenotypes are increasingly being reported all over the world, exposing significant controversies on the present and future role of vancomycin and teicoplanin in the treatment of severe infections sustained by hVISA-MRSA isolates [1]. In these strains, often with vancomycin minimum inhibitory concentrations (MICs) in the 1–2 mg/L range, this reduced susceptibility has been attributed to various cell-wall abnormalities, evolving in a multistep fashion. Even if the genetic occurrence at the base of the hVISA phenotype has not yet been established, these strains often harbor modifications in *gra*SR, *vra*SR and *wal*KR two-component system (TCS) regulatory genes, and RNA polymerase beta subunit (*rpo*B) encoding genes [2].

In this scenario, daptomycin is always used as an alternative option for the treatment of infections caused by *S. aureus*, with a potent bactericidal activity against MRSA, excluding VISA and hVISA clinical strains. Moreover, infections sustained by daptomycin non-susceptible *S. aureus* (DNS) and DNS-VISA phenotypes, even if rare, are increasingly associated with increased higher mortality and morbidity rates [3].

Dalbavancin, a new second-generation semisynthetic lipoglycopeptide, active against Gram-positive pathogens, including MRSA, has recently been approved for the treatment of severe skin infections [4]. The analysis of the bactericidal activity by time-kill curve assays has shown that dalbavancin performs 4–8 times higher activity than vancomycin *versus* MRSA, and its activity, alone and in association, has also been tested against MRSA, VISA, hVISA and DNS isolates [5,6].

The objective of this study was to investigate the in vitro antibacterial activity of dalbavancin against strains of *S. aureus* belonging to diverse phenotypes of antibiotic resistance: methicillin-susceptible and methicillin-resistant *S. aureus* (MSSA, MRSA); vancomycin-susceptible *S. aureus* (VSSA), hVISA and VISA; DNS and rifampicin-resistant (RIF-R) *S. aureus*. All strains were isolated from patients with severe infections (Blood Stream Infections—BSIs, Low Respiratory Tract Infections—LRTIs—and Skin and Soft tissue Infections—SSTIs), as part of a multicenter study conducted in Italy, and molecularly characterized by routine typing methods (sequence-type—ST; Staphylococcal Cassette Chromosome *mec*—SCC*mec*; staphylococcal protein A—*spa* type) [7,8]. We also evaluated bactericidal activity of dalbavancin against a sample of the main representative multidrug-resistant (MDR) and virulent epidemic clones (ST22-SCC*mec*-IVh, ST228-SCC*mec*I RIF-R and ST1-SCC*mec*IV DNS), with increasing antibiotic resistance profiles.

## 2. Results and Discussion

Dalbavancin showed a potent in vitro activity against S. aureus (MIC range ≤ 0.007–0.125 mg/L), with MIC_50_/MIC_90_ values within the susceptibility breakpoints, according to the international guidelines.

Remarkably, its activity was retained against the most refractory MDR-MRSA isolates belonging to the major MRSA clones: ST228-SCC*mec* I, ST8-SCC*mec* IV, ST239-SCC*mec* III, ST5-SCC*mec* II, and ST22-SCC*mec*-IVh. Dalbavancin also demonstrated activity against DNS isolates, making it a valuable tool against these periodically reported strains [7,8,9]. In only two cases we found non-susceptibility values: a hospital-associated HA-MRSA/VSSA strain belonging to the USA500-like (ST8-SCC*mec* IV) clone with a dalbavancin MIC value one dilution above the susceptibility breakpoint (MIC 0.25 mg/L), and a DNS/VISA strain belonging to ST1-VISA-SCC*mec* IV clone, with an MIC value of 2 mg/L (Table 1). This strain was also RIF-R, carrying the most spread N481Y RpoB substitution [8]. The same increase in MIC values was similarly observed in the VISA, Mu50 and NRS402 control strains (Table 2).

The selected RIF-R strains showed the highest percentage of isolates with non-susceptibility to dalbavancin (n.9, 18%), although with MIC values between 0.25 and 0.5mg/L. These strains showed nearly all a hVISA phenotype and belonged to the most spread Italian clone ST228-SCC*mec* I-*spa*-type t001/t041 clone (7 out of 9), and to ST5-SCC*mec* II-*spa*-type t002 clone (1 out of 9), the same as the VISA/hVISA controls (Mu50/Mu3) included in the study, with which they share common characteristics including a thickened cell wall [10]. Only one RIF-R/VSSA strain, showing a one-fold higher dalbavancin MIC value, belonged to ST8-SCC*mec* IV*/spa*-type t008 clone usually spread in the community setting. In the interpretation of this result, which deserves further insights, it should be taken into account that the rifampicin-resistant phenotype of these strains occurred from different mutations in the gene encoding RNA-polymerase (*rpo*B), whose alteration has been associated with multiresistant daptomycin, vancomycin and beta-lactams phenotypes [8,9].

The results of time–kill curve assays provided a dynamic picture of the bactericidal activity against three model strains: the analyses were conducted with different concentrations of dalbavancin, equal to the MIC values 2, 4 and 8 times higher than the MIC value, respectively.

**Figure 1:**
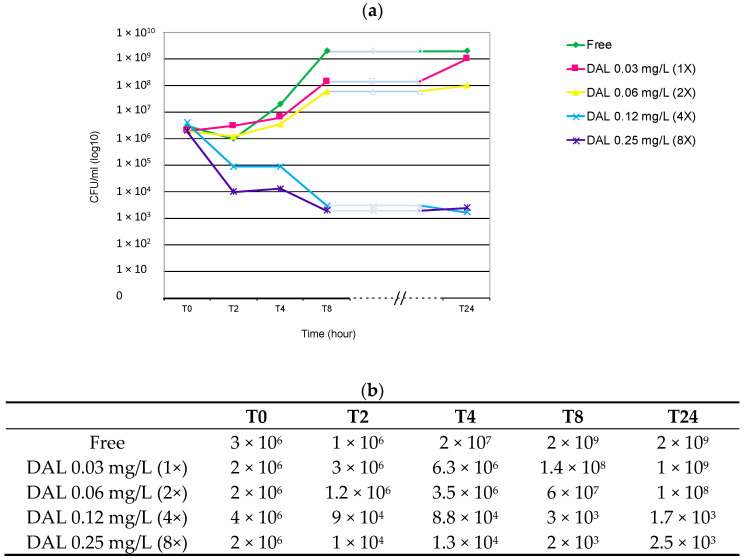
E-MRSA15 ST22-IVh-t223 MIC (1X) 0.03mg/L: (**a**) Graph of curves obtained. (**b**) Average of duplicate viable counts (CFU/mL) observed at T0-T2-T4-T8 and T24 time intervals and after exposure to different concertation of dalbavancin (1-2-4 and 8X MIC). DAL: dalbavancin. Time (hour): 0, 2, 4, 8 and 24 h after the starting *inoculum*. The red row represents the threshold of bactericidal activity (≥3 log_10_ decrease).

Dalbavancin exerted a potent bactericidal activity against the HA-MRSA/VSSA strain belonging to the E-MRSA15-ST22-SCC*mec*-IVh *spa*-type t223 clone after 8h from the starting *inoculum* at concentrations of 0.12 and 0.24 mg/L. Dalbavancin concentrations of 0.03 and 0.06 mg/L were not sufficiently bactericidal, therefore bacterial growth increased over time, miming the antibiotic-free control (Figure 1).

**Figure 2:**
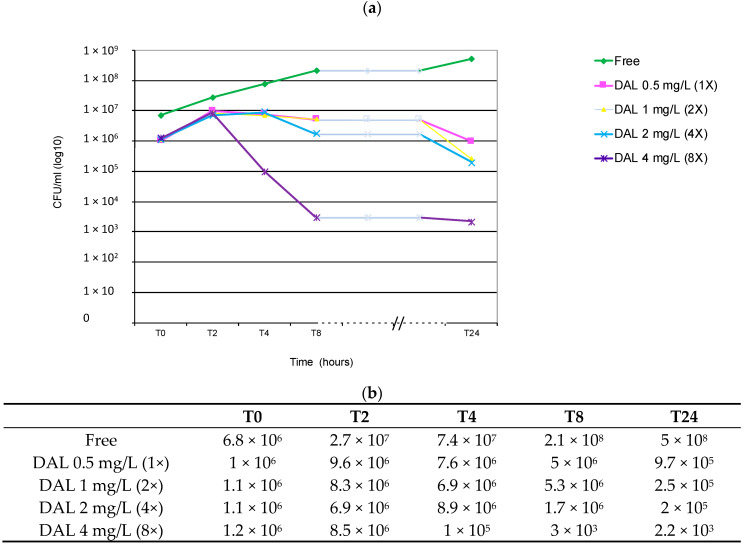
MRSA RIF-R ST228-I-t041 MIC (1X) 0.5 mg/L. (**a**) Graph of curves obtained. (**b**) Average of duplicate viable counts (CFU/mL) observed at T0-T2-T4-T8 and T24 time intervals and after exposure to different concentration of dalbavancin (1-2-4 and 8X MIC). DAL: dalbavancin. Time (hour): 0, 2, 4, 8 and 24 h after the starting *inoculum.* The red row represents the threshold of bactericidal activity (≥3 log_10_ decrease).

Against the RIF-R/hVISA strain belonging to ST228-SCC*mec* I *spa*-type t041, dalbavancin bactericidal activity was exhibited at the higher concentration assayed of 4 mg/L (8X MIC) at 8 and 24 h intervals, and a non-bactericidal reduction of only 1 log_10_ at lower concentrations (1 and 2 mg/L), at 24 h (Figure 2).

**Figure 3:**
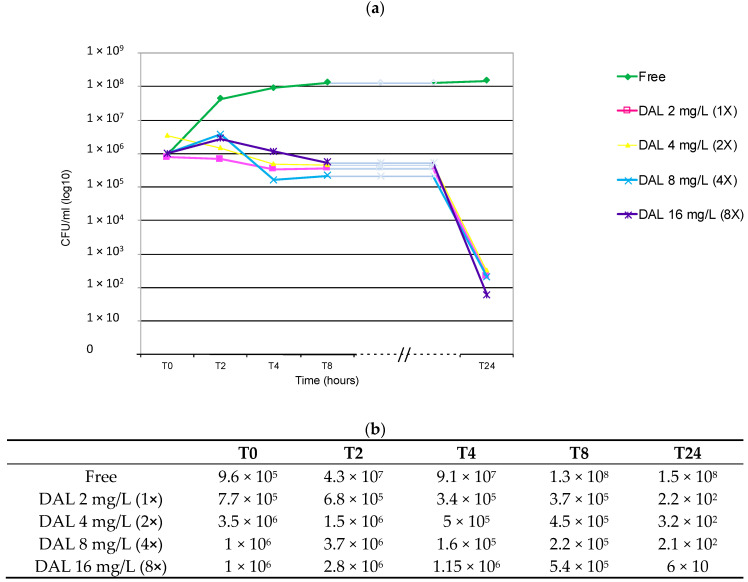
MRSA DNS ST1-IV-t386 MIC (1X) 2 mg/L. (**a**) Graph of curves obtained. (**b**) Average of duplicate viable counts (CFU/mL) observed at T0-T2-T4-T8 and T24 time intervals and after exposure to different concertation of dalbavancin (1-2-4 and 8X MIC). DAL: dalbavancin. Time (hour): 0, 2, 4, 8 and 24 h after the starting *inoculum*. The red row represents the threshold of bactericidal activity (≥3 log_10_ decrease).

The time–kill curve assay showed a stronger bactericidal activity of dalbavancin against the DNS strain belonging to ST1-SCC*mec* IV *spa*-type t386, at 24 h and at all the concentrations assayed (Figure 3). The bacterial growth considerably decreased from 3–4 log_10_, in the presence of 1X-2X and 4X MIC, to 5 log_10_ with the highest dalbavancin concentration (16 mg/L—8X MIC). In this clone, the bactericidal activity was exhibited only after 24 h from the starting *inoculum*. These data therefore deserve an in-depth analysis, aimed at understanding if a correlation between daptomycin non-susceptibility and delayed but conclusive bactericidal activity is conceivable [11,12].

## 3. Methods Section

A total of 124 strains of *S. aureus* selected from a large collection of isolates from 63 centers distributed throughout Italy—as part of the multicentre study CoSA-AMCLI 2012 [7,8]—were tested for susceptibility to dalbavancin according to standard methods [13,14]. Characterization of VSSA/hVISA/VISA phenotypes were also assessed by population analysis assay (PAP/AUC), following previously published procedures [15]. All isolates were already genetically characterized by PFGE, SCC*mec*-typing, Multilocus Sequence Typing (MLST—https://pubmlst.org/organisms), *spa*-typing (https://spaserver.ridom.de), presence of *pvl* gene and evaluation of *rpo*B mutations responsible for the RIF-R phenotype, as previously published [7,8].

In particular, the sample consisted of n. 23 MSSA; n. 24 MRSA/VSSA; n. 22 MRSA/hVISA; n. 5 DNS/MRSA and a selected sample n. 50 RIF-R/MRSA. Two VISA (Mu50 and NRS402) and two hVISA (Mu3 and NRS22) strains, a vancomycin-resistant (VRS1) strain and an MSSA (ATCC 29213) strain were included as controls.

Dalbavancin in vitro activity was tested by a microdilution method. For the preparation of dalbavancin, 100 mg of powder was completely dissolved in 10 mL Dimethyl sulfoxide (DMSO Sigma-Aldrich-Merck KGaA, Darmstadt, Germany). Microtiter plates were prepared with 100 µl of Mueller Hinton Broth, Cation-adjusted (CAMHB, NutriSelect™ Plus, Becton Dickinson, Franklin Lakes, NJ, USA), in which 100 µl of antibiotic were added at scalar concentrations starting from an initial concentration of 8 mg/L. For dalbavancin, 0.002% polysorbate-80 (Tween 80) (Merck, Darmstadt, Germany) was previously added to the broth CAMHB medium [13,14]. A standard *inoculum* of 0.5 McFarland was used as described by the CLSI M07-A10 document [16] and the results interpreted according to the European Committee on Antimicrobial Susceptibility Testing (EUCAST) breakpoint criteria [10].

The bactericidal activity of dalbavancin was evaluated by time–kill curves, according to standard procedures [17]. Briefly, the experiments were performed in duplicate in 20 mL tubes containing Cation-adjusted Mueller-Hinton broth (CAMHB), NutriSelect™ Plus, Becton Dickinson, Franklin Lakes, NJ, USA) using a starting *inoculum* of 10^5^–10^6^ CFU/mL, with dalbavancin (1X, 2X, 4X and 8X MIC) supplemented with 0.002% Tween 80. Additionally, 100 µl serial dilutions were plated in Mueller Hinton Agar 2 (MH agar 2, NutriSelect™ Plus, Becton Dickinson, Franklin Lakes, NJ, USA), in different time intervals T0-T2-T4-T8 and T24 (0, 2, 4, 8 and 24 h) and after overnight incubation at 37 °C the grown colonies were counted. All experiments were repeated at least three times, and results of a representative experiment are presented. Killing curves were constructed by plotting the log_10_ CFU ml−1 *versus* time over 24 h, and the change in bacterial concentration was determined. Data points are averages from duplicate viable count determinations (CFU/mL) within an experiment. Bactericidal activity was defined as a reduction of 99.9% (≥3 log10) of the total number of CFU/mL of the starting *inoculum* (10^5^–10^6^ CFU/mL)*,* after 24 h of exposure with the antibiotic. Bacteriostatic activity was defined as maintenance of the starting *inoculum* or a reduction of less than 99.9% (<3 log10) of the total number of CFU/mL of the starting *inoculum* [17].

## 4. Conclusions

Our study underlined the excellent in vitro antibacterial and bactericidal activity of dalbavancin against representative strains belonging to the major epidemiologically diffused phenotypes, including MRSA/hVISA, DNS and RIF-R strains, confirming the stability of its potency against *S. aureus* isolates [18]. MRSA strains showing heteroresistance to vancomycin (hVISA), often with vancomycin MICs in the 1–2 mg/L range, are increasingly being reported and a systematic review of the literature on hVISA reported that patients infected with these organisms had a 2.37-fold greater failure rate compared to those infected with fully susceptible (VSSA) organisms [19]. Consequently, significant controversy exists regarding the current and future roles of vancomycin and teicoplanin in the treatment of serious hVISA-MRSA infections. Our data corroborate with what has been recently reported by other authors, reinforcing the hypothesis that dalbavancin may be a valuable agent against problematic pathogens [6,7,8,9,10,11,12,13,14,15,16,17,18,19,20]. The interpretation of the slightly higher rate of dalbavancin non-susceptibility among RIF-R/hVISA isolates needs further investigations, although it is possible to assume that the presence of *rpo*B mutations in these strains [8], already associated with the emergence of vancomycin-intermediate resistance, may affect the antimicrobial activity. The major refractoriness of RIF-R/hVISA and DNS strains is also corroborated by other expression studies conducted on VISA and hVISA, in which the drastic change in the cell transcriptional profile was demonstrated to be mainly associated to *rpo*B mutations [21]. Nonetheless, it is to be mentioned that the dalbavancin MICs of these strains were only one/two dilutions above the EUCAST breakpoint, and that many in vitro and in vivo preclinical studies predicted that the pharmacokinetic/pharmacodynamic (PK/PD) profiles usually persist above the MIC level [22]. Our observations suggest that dalbavancin will be considered an excellent therapeutic alternative for the management of severe *S. aureus* infections sustained by MDR strains sharing diverse and increasing behaviors of antibiotic resistance, also belonging to most refractory MRSA phenotypes.

## Figures and Tables

**Table 1:** In vitro activity of dalbavancin *versus* methicillin-susceptible *Staphylococcus aureus* (MSSA) and methicillin-resistant *Staphylococcus aureus* (MRSA) (vancomycin-susceptible *S. aureus* (VSSA), vancomycin-intermediate *S. aureus* (hVISA) and vancomycin-intermediate *S. aureus* (VISA); daptomycin non-susceptible (DNS) and rifampicin-resistant (RIF-R) represented as MIC range (mg/L), MIC_50_/MIC_90_ and n/% of resistant isolates (R).

Strains	n. of Strains	MIC Range (mg/L)	MIC_50_ (mg/L)	MIC_90_ (mg/L)	n. −% (R)
MSSA	23	≤0.007–0.125	0.03	0.125	0
MRSA/VSSA (HA/CA-MRSA)	25	0.015–0.25	0.06	0.125	1 (4%)
MRSA/hVISA (HA/CA-MRSA)	22	≤0.007–0.125	0.06	0.125	0
MRSA/DNS (hVISA + VISA)	4 (3hVISA + 1VISA)	0.06–2	0.06	0.125	1 (25%)
MRSA/RIF-R (hVISA + VSSA)	50 (31hVISA + 19VSSA)	0.015–0.5	0.125	0.25	9 (18%)
**Tot *S. aureus***	124	≤0.007–2	0.06	0.125	11 (8.8%)

MSSA, methicillin-susceptible *S. aureus*; MRSA, methicillin-resistant *S. aureus;* VSSA, vancomycin-susceptible *S. aureus*; hVISA, hetero-resistant vancomycin-intermediate *S. aureus*; DNS, daptomycin non-susceptible; RD-R, rifampicin-resistant. MIC range (mg/L) refers to the lower and higher dalbavancin MIC values; EUCAST dalbavancin clinical breakpoint R > 0.125mg/L.

**Table 2:** In vitro activity of dalbavancin *versus S. aureus* control strains.

Subcategory	Strain	ST-MRSA-SCC*mec*	*spa*-Type	Dalbavancin MIC (mg/L)
MSSA	ATCC29213	-	-	0.06
MRSA-hVISA	Mu3	ST5-hVISA-II	t002	0.125
MRSA-VISA	Mu50	ST5-hVISA-II	t002	1
MRSA-hVISA	NRS22	ST45-hVISA-II	t266	0.03
MRSA-VISA (DNS)	NRS402	ST5-VISA-II	t002	1
MRSA-VRSA	VRS1	ST5-VRSA Tn*1546 van*A	-	≥4

Clone characterization by means of: ST—Sequence Type; SCC*mec*—Staphylococcal Cassette Chromosome *mec*; *spa* type—staphylococcal protein A.
